# Systems medicine disease maps: community-driven comprehensive representation of disease mechanisms

**DOI:** 10.1038/s41540-018-0059-y

**Published:** 2018-06-02

**Authors:** Alexander Mazein, Marek Ostaszewski, Inna Kuperstein, Steven Watterson, Nicolas Le Novère, Diane Lefaudeux, Bertrand De Meulder, Johann Pellet, Irina Balaur, Mansoor Saqi, Maria Manuela Nogueira, Feng Q. HeFeng, Andrew Parton, Nathanaël Lemonnier, Piotr Gawron, Stephan Gebel, Pierre Hainaut, Markus Ollert, Ugur Dogrusoz, Emmanuel Barillot, Andrei Zinovyev, Reinhard Schneider, Rudi Balling, Charles Auffray

**Affiliations:** 1 European Institute for Systems Biology and Medicine, CIRI UMR5308, CNRS-ENS-UCBL-INSERM, Université de Lyon, 50 Avenue Tony Garnier, 69007 Lyon, France; 2 Luxembourg Centre for Systems Biomedicine (LCSB), University of Luxembourg, Campus Belval, 7 Avenue des Hauts-Fourneaux, L-4362 Esch-sur-Alzette, Luxembourg; 3 Institut Curie, Paris, France; 4 INSERM, U900 Paris, France; 5 Mines ParisTech, Fontainebleau, France; 6 PSL Research University, Paris, France; 7 Northern Ireland Centre for Stratified Medicine, Ulster University, C-Tric, Altnagelvin Hospital Campus, Derry, Co Londonderry, Northern Ireland, BT47 6SB UK; 8 The Babraham Institute, Babraham Research Campus, Cambridge, CB22 3AT UK; 9 Department of Infection and Immunity, Luxembourg Institute of Health (LIH), House of BioHealth, 29 Rue Henri Koch, L-4354 Esch-Sur-Alzette, Luxembourg; 10 Institute for Advanced Biosciences, University Grenoble-Alpes-INSERM U1209-CNRS UMR5309, Site Santé – Allée des Alpes, 38700 La Tronche, France; 11 Department of Dermatology and Allergy Center, Odense Research Center for Anaphylaxis, University of Southern Denmark, Odense, Denmark; 12 Faculty of Engineering, Computer Engineering Department, Bilkent University, Ankara, 06800 Turkey

**Keywords:** Diseases, Systems biology, Scientific community

## Abstract

The development of computational approaches in systems biology has reached a state of maturity that allows their transition to systems medicine. Despite this progress, intuitive visualisation and context-dependent knowledge representation still present a major bottleneck. In this paper, we describe the Disease Maps Project, an effort towards a community-driven computationally readable comprehensive representation of disease mechanisms. We outline the key principles and the framework required for the success of this initiative, including use of best practices, standards and protocols. We apply a modular approach to ensure efficient sharing and reuse of resources for projects dedicated to specific diseases. Community-wide use of disease maps will accelerate the conduct of biomedical research and lead to new disease ontologies defined from mechanism-based disease endotypes rather than phenotypes.

## The concept

### Disease mechanisms in the context of translational medicine projects

Large amounts of high-throughput data are routinely generated in an effort to better understand diseases, adding to our extensive and diverse biomedical knowledge. Common objectives include the identification of disease biomarkers, molecular mechanisms, potential drug targets and disease subtypes for better diagnostics and stratification of patients.^1^

Using such diverse and complex high-throughput datasets to meet the current and future demands of research in basic and translational medicine is challenging. Our experience in large-scale translational medicine projects (Supplementary material S1) is that the difficulties associated with such tasks are often vastly underestimated. When it comes to disease-specific functional analysis and systematic data interpretation, computational and mathematical tools have not developed at the same pace as laboratory technologies. Interpreting data in a given context still mainly relies on statistical approaches, e.g., pathway enrichment analysis. To advance beyond context-independent use of canonical pathways, dedicated knowledge maps are needed, which would provide the molecular mechanisms involved in given diseases.

Charting maps, from geography to anatomy, is an essential scientific activity in many fields. Maps do not only chart a territory but also facilitate our understanding.^2^ A mechanistic representation was first applied on a large scale to metabolic pathways in the form of the wall charts created by Nicholson^3^ and Michal.^4^ Mechanistic representation of extensive signalling pathways was pioneered by Kurt Kohn^5^ and Hiroaki Kitano^6^ and developed into the Systems Biology Graphical Notation (SBGN) standard.^7^

**Fig. 1:**
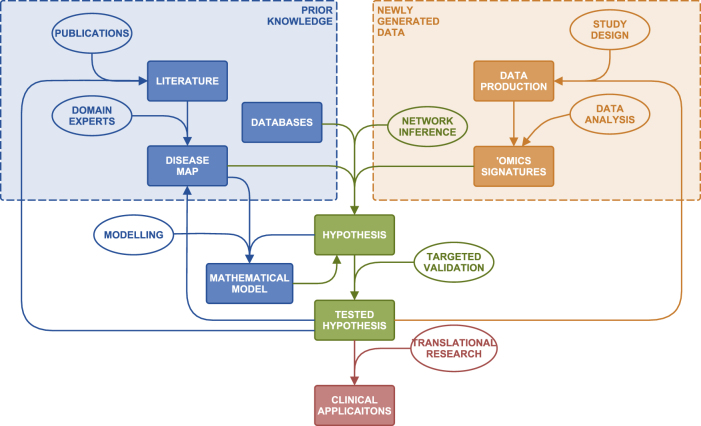
Outline of the systems medicine rationale. The diagram represents the transformation of diverse prior knowledge and newly generated data into hypotheses using computational and mathematical methods, tools and approaches appropriate for each step

In order to bridge knowledge maps and the big data of health-care research, we have engaged in the development of highly detailed and specific representations of known disease mechanisms (Table 1).^8–10^ Having these resources, we employed complementary techniques that use prior knowledge for data and network analysis and hypothesis generation^11^ in systems medicine projects (Fig. 1).

This paper describes the concept of a disease map, a resource that focusses on disease mechanisms and can be used to develop computational models of disease with advanced data interpretation methods. We put forward the view that disease maps should be developed through a community effort that facilitates collaborative research in support of translational medicine projects.

**Table 1:** Comparison of published disease maps

Feature	AlzPathway	Parkinson’s disease map	ACSN
Webpage	http://alzpathway.org	http://pdmap.uni.lu	https://acsn.curie.fr
Online exploration	Payao (Apache Flex)	MINERVA (Google Maps API)	NaviCell (Google Maps API)
Content development	CellDesigner^a^	CellDesigner^a^	CellDesigner^a^
Standard formats	SBML, BioPAX	SBML	SBML, BioPAX
Number of nodes	1538	5073	5975
Number of processes	1127	2108	4826
Number of proteins	721	2973	2371
Number of metabolites	300	703	595
Number of genes	33	202	159
Number of references	>100 Review articles	1307	2919

Disease maps represent hallmark pathways and processes associated with the disease: Alzheimer’s disease,^8,54^ Parkinson’s disease^9^ and cancer.^10,55^ The three disease maps are the result of manual curation of the relevant literature and provide a manually drawn layout using CellDesigner (http://www.celldesigner.org). The exploration of the maps is available through Payao,^56^ MINERVA^27^ and NaviCell.^28,29^

^a^CellDesigner supports graphical notation and symbols based on the Systems Biology Graphical Notation^7^ Process Description language Level 1 draft as of May 2008 (http://www.celldesigner.org/features.html). To visualise diagrams in the current version of the SBGN standard (http://sbgn.org) it is possible to use the CellDesigner SBGN Viewer (http://www.celldesigner.org/help/)

### Disease map definition

**Fig. 2:**
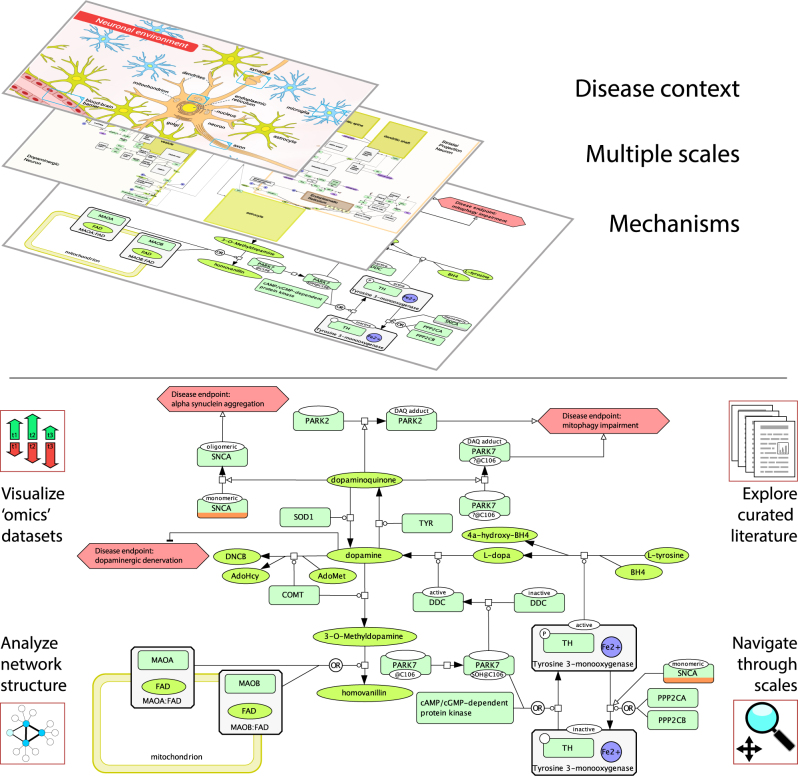
A fragment of the Parkinson’s disease map. **a** A disease map repository allows visual multiscale exploration of the contextualised, disease-relevant mechanisms. Here, the contents of the Parkinson’s Disease map can be explored across scales from the neuronal environment to the detailed molecular pathobiology of the disease. Higher scales emerge from the underlying mechanistic descriptions. **b** Mechanistic details of dopamine metabolism in the dopaminergic neurones are shown. The involvement of Parkinson’s disease familial genes, PARK2 (Parkin), PARK7 (DJ-1) and SNCA (alpha-synuclein) is highlighted. Excessive oxidation of dopamine into dopamine quinone may lead to conformational changes in proteins and subsequently result in molecular neuropathology associated with Parkinson’s, marked as “disease endpoints” in the figure

A disease map can be defined as a comprehensive, knowledge-based representation of disease mechanisms. Essentially, it is a conceptual model of a disease. It contains disease-related signalling, metabolic and gene regulatory processes with evidence of their relationships to pathophysiological causes and outcomes^8–10^ (Fig. 2). To describe the complexity of a disease it is important to capture not only biochemical interactions but also physiological mechanisms.

A disease map is represented graphically and is encoded in a standard computer-readable and human-readable format, allowing its transformation, partially or wholly, into mathematical models for predictive analysis. We specifically distinguish the literature-derived conceptual model of a disease and the different types of mathematical models that can be produced from it. This way we make the core resource updatable and sustainable while enabling the production of various models by adding assumptions, hypothetical mechanisms and model parameters. The proposed new mechanisms are then confirmed or rejected, and the conceptual core model updated accordingly.

Development of disease maps relies on an active involvement of domain experts. In contrast to most knowledge management solutions that directly reuse available information stored in various databases, building disease maps requires to actively look for mechanistic details and add missing pieces.

The resulting representations provide curated systems-level views of the mechanisms associated with a given disease for interpretation by biomedical experts as well as a broader audience, e.g., physicians, teachers and students.

Multiscale knowledge management is at the heart of the disease map concept. This means developing and exploiting protocols for the high-quality representation of information at different levels of granularity including subcellular, cellular, tissue, organ and organism levels. Although we have become better at describing biological events, there are still challenges to be faced, such as the representation of the physiological layers and the inclusion of regulatory mechanisms. Describing diseases in standardised formats will simplify cross-disease comparisons and will facilitate the identification of similarities and differences in molecular and cellular modules between diseases.

### The challenges in assembling disease maps and suggested solutions

#### Determining the content

Taking into account the high level of connectivity in biological networks, potentially many biological processes can be linked to or affected by a disease. One of the first steps in a disease map construction is therefore setting the scope: defining hallmark molecular mechanisms and affected tissues and drafting their relationships. From this step onwards the curators of a disease map should consult domain experts. Community-driven expert-approved construction is the best strategy to build a trusted resource. The developed conceptual model of a disease can be further refined in the cycle of producing and then confirming or rejecting hypotheses.

#### Involving domain experts

The major challenge of offering a consensus view is creating a community of leading domain experts to participate actively in the development of each disease map. The experience of the already published maps ensures that this can be done successfully. For example, the Atlas of Cancer Signalling Networks (ACSN) and the Parkinson’s Disease Map continuously work with dozens of experts and organise regular dedicated workshops (https://wwwen.uni.lu/lcsb/research/parkinson_s_disease_map/expert_curation, https://acsn.curie.fr/about.html). Domain experts are joining because they are motivated not only by their own research interest, but also by the possibility of storing and sharing their experience and concepts across multiple diseases that may have common underlying molecular bases, and by the benefits a description of disease mechanisms can bring to the scientific community and patients. In many cases clinicians themselves initiate the development of such resources. This high-quality approach is assimilated by every project that joins the effort. Best practices are being further refined and currently the process assumes working on an overview diagram where the main hallmarks are being defined and also on clarifying specific focussed detailed mechanisms important for disease progression.

#### Complexity management

With a growing body of literature, a disease map is becoming increasingly extensive and complex. Many of the published disease map resources^8–10,12^ first worked on smaller sub-maps before creating a large single map. We would like to build on this experience of working with sub-maps and explore the idea of hierarchically structured maps learning from the approach used in Reactome.^13^ This way, instead of constantly extending a single map, we can offer an interactive navigation through the multilayer network using comparatively small easy-readable, easy-updatable and often reusable sub-maps.

#### Manually designed diagrams

One of the major time-consuming tasks is manually designing human-readable maps. The layout of such maps reflects a biological concept in an easily-comprehensible form. The currently available algorithms for automatic layout are still insufficient for competing with human-made maps. Solving this problem would offer new opportunities and noticeably improve the development and management of large-scale biological networks.

## The Disease Maps Project

### A community of communities

Disease map generation and curation require an extremely broad and deep domain knowledge and is considerably resource intensive. The development of such maps to an acceptable level of rigour and interoperability, the assembly of the complementary computational and mathematical tools require significant physiological and biomedical expertise combined with analytical skills. Hence, an activity of this scale and scope can only be undertaken as a synergistic effort of many communities dedicated to specific diseases. The members of these communities need to agree on working according to an approved set of standards for curation, representation and accessibility and to develop complementary tools that exploit the data captured with these standards.

The following themes are fundamental to this large-scale collaborative network.

**Fig. 3:**
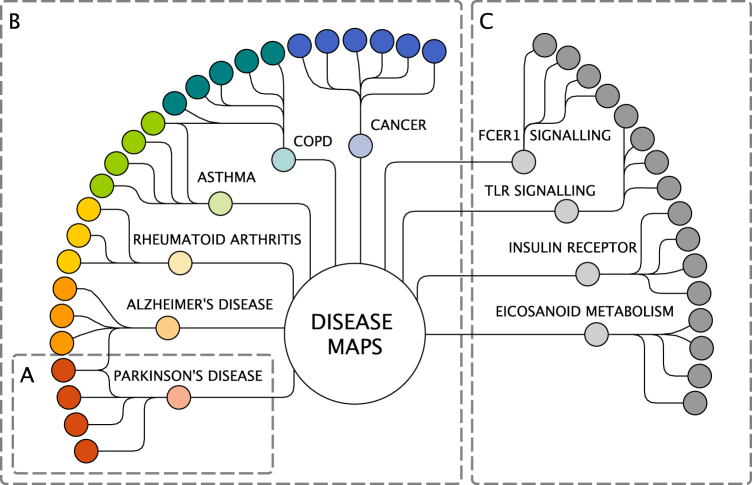
The Disease Maps Project as a community of communities. **a** A collaboration for building one disease map. **b** Disease expert groups. **c** Pathway expert groups. Light colours: computational biology groups. Solid colours: domain experts. The Disease Maps hub is to be used for sharing experience, improving best practices and agreed-upon protocols, exchanging reusable biological processes and pathway modules. It is also an effort to create an infrastructure and set of tools to help each project to progress faster
- 2. These *disease* expert *groups* are brought together in order to enable sharing and exchange of expertise and best practices (Fig. 3b). For example, different chronic diseases are likely to have common inflammatory mechanisms and those groups would benefit from working together.
- 3. Another layer of the network includes *pathway expert groups*, each focussed on particular biological processes or pathway modules (Fig. 3c). We anticipate the emergence of a new type of high-quality database in which reference pathways would be developed and systematically updated by the best experts in the respective fields, and in which the role of pathways can be explored across diseases.
- 4. Support of a larger scientific community of interconnected projects in order to advance the required technologies and avoid duplicated activities.

- 1. A *disease* *expert group*. A total of 5 to 20 domain experts (clinicians, experimental biologists) contribute to each disease map (Fig. 3a) and collaborate with the computational biologists and curators who develop the map.

In order to address these challenges proactively and in a sustainable manner, the Luxembourg Centre for Systems Biomedicine, Institut Curie and the European Institute for Systems Biology and Medicine have initiated the formation of a collaborative network, the “Disease Maps Project” (http://disease-maps.io), that takes an active role in the development of disease maps by fostering knowledge exchange and integrating the activities into a community effort. The first face-to-face community meeting (http://disease-maps.io/events) included groups from six countries developing disease maps for anaphylaxis, asthma, atherosclerosis, cystic fibrosis, cancer, multiple sclerosis, Parkinson’s disease and rheumatoid arthritis (http://disease-maps.io/projects). Other groups then joined, developing maps on, e.g., acute kidney injury and coronary artery disease. The second community meeting was focussed on mathematical modelling, integrating resources and coordinating activities with friendly projects.^14^ Additional activities are in preparation and the list of maps is continuously growing.

### Expected development of the project

The Disease Maps Project builds on the research interests of many groups in understanding the mechanisms of particular diseases. This ensures its natural expansion and efficiency: saving time and resources by sharing tasks and investing together in the development of the required tools and pipelines. We anticipate that many disease maps will be initiated and supported within the next several years, which will transform into new technologies tested and made available for rapid advances in translational research.

One of the important driving forces that bring separate groups together is the interest in studying disease comorbidities and shared mechanisms. In addition, we put emphasis on initiating clusters of disease maps for diseases that are shown to have similar mechanisms: allergic diseases, autoimmune diseases, neurodegenerative disorders and cancers of different origins and types. For example, we aim to investigate common mechanisms of asthma (ongoing effort, advanced stage), allergic rhinitis (planning stage) and atopic dermatitis (planning stage).

Agreements on collaboration and developing technologies (http://disease-maps.io/relatedefforts) are reached with complementary efforts such as WikiPathways^15^ (https://wikipathways.org), Pathway Commons^16^ (http://www.pathwaycommons.org), the Physiome Project^17^ (http://physiomeproject.org), Virtual Metabolic Human^18^ (https://vmh.uni.lu) and the Garuda Alliance (http://www.garuda-alliance.org). In each case, we identified overlapping activities and directions of research. Specific common interests are standard formats, interoperability among pathway resources, reuse of pathway modules (WikiPathways, Pathway Commons), description and modelling of physiological and pathophysiological mechanisms (Physiome), linking metabolic and signalling networks (Virtual Metabolic Human) and pipelines and communication between tools (Garuda). Starting from small focussed collaborative projects, we plan to establish common frameworks and aim to progress together as partners while optimising the use of resources and the expertise.

Relationships between normal and disease pathways should be further explored. Developing a disease map often means that (1) pathway information is reused and contextualised for a specific condition and cell type, or/and (2) the pathway structure is modified by a disease. The structure of a pathway often remains the same but the concentrations of the involved proteins and metabolites change significantly as the disease progresses; for example, the FcεRI activation by IgE and allergen in asthma leads to the production of eicosanoids.^19^ In other cases, for instance in cystic fibrosis, it is mainly about modified or interrupted pathways when a mutation causes an altered life cycle of the CFTR protein.^20^ Leveraging non-disease pathway resources, flexible navigation within normal and altered-by-disease pathways, the ability to compare the healthy and disease state would be important for the future advances of the Disease Maps Project. There is a need for a technology for comparing pathway modules, determining what contributes to a well-developed reconstruction, on-the-fly decision-making for choosing an appropriate module for a particular disease map.

The Disease Maps Project also aims to align its work with the European-wide research infrastructures roadmap (http://www.esfri.eu/roadmap-2016), e.g., as part of CORBEL (Coordinated Research Infrastructures Building Enduring Life-science Services; http://www.corbel-project.eu).

### Integrating resources

Prior to the Disease Maps initiative, the published reconstructions^8–10,12^ used different ways to deliver their maps to users. To review several projects, users would have to go to individual project pages and, in most cases, use different systems employed for map browsing. During our second community meeting we started discussing the exploration of disease maps via web-platforms and integration of maps in a shared repository.^14^

The first step for integrating various disease maps is agreeing on shared formats as the common ground for all the involved projects. The maps should be available in the established standards such as SBGN, systems biology markup language (SBML)^21^ and BioPAX.^22^ Neo4j graph database is another framework to apply as it is well suited for queries via its declarative Cypher language. The graph database approach has been shown to facilitate management and exploration of biomedical knowledge.^23^ Neo4j format is used, for example, for Recon2^24^ and Reactome.^25^ With this prospect in mind, we have developed a tool for converting SBGN-ML to Neo4j format.^26^

Reusable disease map modules can be stored in pathway-oriented databases. A solution can be found in collaboration with Pathway Commons,^16^ WikiPathways^15^ and Reactome^25^ while minimising duplicated activities and addressing compatibility issues. The disease maps themselves should be available for browsing and exploration in a unified way via a centralised easily accessible web repository using platforms such as MINERVA,^27^ NaviCell^28,29^ and iPathways+^12^ (http://www.ipathways.org/plus).

### The pillars of the community effort

The key aspect of the Disease Maps Project is the interoperability between various disease maps. An agreement on common guidelines and standards will enable projects to help each other, share common tasks and promote trust in the quality of content provided by another group.

We propose to build this community effort upon the following guiding principles.

#### Open access

We believe that this is an essential principle supporting the interests of each of the individual groups as well as industrial partners. Benefits derive from applying the disease maps to specific questions, not from the ownership of the reference maps. Open access invites advanced domain expertise on a larger scale and provides a better chance of developing a trusted reference resource for a particular disease.

#### Standard formats

**Fig. 4:**
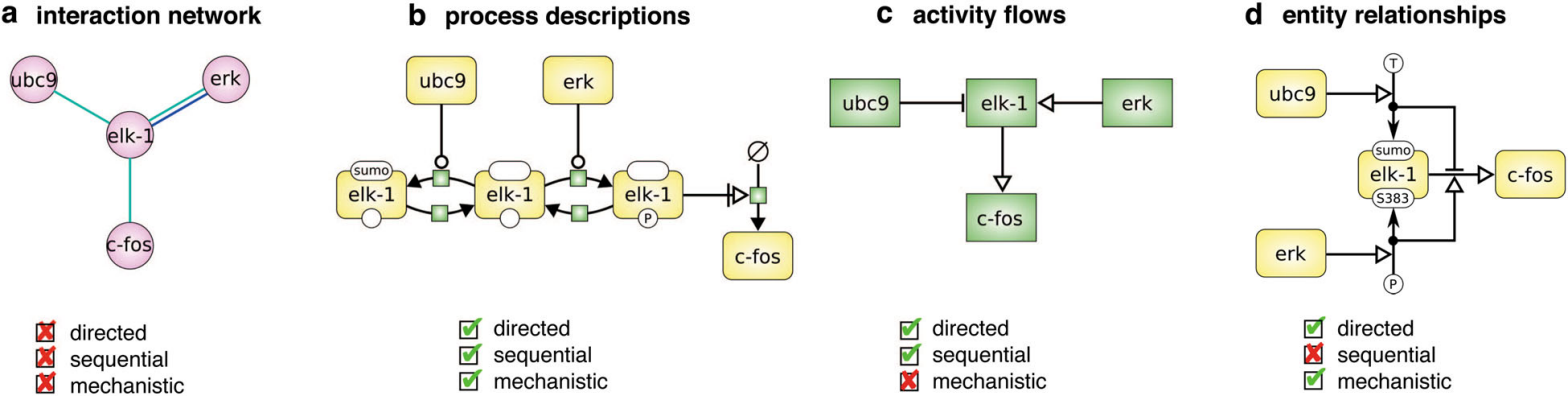
Representation of biological networks. Note that in all the four cases (**a**−**d**) the same set of proteins is shown but the relationships are represented differently. The disease maps employ the two sequential representations: process descriptions and activity flows. These representations correspond to the Process Description and Activity Flow languages of the SBGN standard (adapted from ref. ^53^)

Efficient knowledge exchange derives from the use and development of standardised formats. Specific visual formats for disease maps are the SBGN Process Description and Activity Flow languages (Fig. 4).^7,30^ The maps should be made accessible in a variety of formats including SBML,^21^ BioPAX^22^ and Neo4j.^26^ The recently published System Biology Format Converter^31^ makes the task of communication between different formats much simpler.

#### Modular approach

Process information in disease maps is organised as reusable modules and sub-networks. Modularity facilitates exchange of disease map content and distributed curation between groups. Another advantage, often overlooked, is the possibility of having alternative versions, for instance with different emphasis or granularity. Using modules allows the networks to be extended almost indefinitely while keeping the system manageable.

#### Consistent quality

Shared protocols and guidelines help improve the quality of the representations, in particular via consistency. Credibility and adequate knowledge representation is a major challenge in representing molecular mechanisms of a disease.

#### Best practices

The community approach and efficient communication play an important role: new technological advances developed by one group become immediately accessible to all the participants.

### Required resources, infrastructure and tools

One of the primary requisites for the Disease Maps Project is a collection of biological process and pathway modules that can be reused across maps. The focus could be on, e.g., common components of inflammation, immune responses and metabolism due to their pervasive nature in health and disease.

The process of map creation becomes more distributed and offers new challenges for tool developers.^14^ With the advances in cloud environments and web technologies, it is expected to have an easy access to drawing maps online, and the development of the web-based Newt Editor (http://newteditor.org) is a promising step in that direction. Also, it becomes critical to have collaborative editing capabilities similar to Google Docs (https://www.google.com/docs/about), version control similar to Git (https://git-scm.com/about) and support for crowdsourcing as successfully implemented by WikiPathways.^15^ Advanced layout algorithms are essential for further progress in semi-automatic map construction and on-demand visualisation of complex systems.^32,33^ We encourage the development of new tools as open-source software to allow many groups to simultaneously contribute and extend existing functionalities.

To accommodate the requirements of the activities and tasks within the community project, it is important to balance the use of the well-established intensively used software and a step-by-step exploration to the desired new functionalities. As all the published disease maps^8–10,12^ are created in CellDesigner, any new tools need to ensure compatibility with such existing format and provide the necessary interoperability.

Of particular importance are the issues of browsing and semantic zooming^29^ through each disease map. The word “map” implies the possibility of navigation and a “geographic-like” view of molecular interactions. Generation of new hypotheses requires capabilities for high-dimensional data overlay and tools that help transform and analyse interaction networks on-the-fly. Extensive representations that include thousands of entities require improved approaches for complexity management, e.g., organised maps and semantic zooming to efficiently manage the content online via such tools as MINERVA^27^ and NaviCell.^28,29^

Disease maps should be integrated with gene- and protein-interaction-based approaches^34–41^ allowing further exploration beyond known disease mechanisms to suggest new disease-associated genes, processes and functional modules, and propose unknown mechanisms.

### Target users

The maps are primarily designed for computational biologists to be applied in the field of biomedical research. We also believe that the disease maps can be used to teach molecular biology of diseases to students. Some promising efforts have been made in this direction among the community members. For example, the Parkinson’s disease map is used for teaching at the University of Luxembourg. The visual components of the maps are especially appealing for explaining the complexity of the modelled disease mechanisms to non-bioinformaticians. Medical doctors are intensively involved in the development of disease maps. It is the experience of the community members that the maps facilitate a productive communication with physicians on disease mechanisms in conjunction with interpretation of experimental data.

At the same time, in their current form, the available maps are not as easily accessible as we would like them to be. The amount of details can be overwhelming if only the comprehensive view is used. As discussed above, we work on presenting disease mechanisms at different layers of granularity, with semantic zooming through the layers. Each layer is essentially a user interface for particular groups of users. The design is to be shaped by the target groups. The community is aware of the necessity to maintain the dialogue with a broader audience to improve the content and the tools.

## Ongoing advances and future perspectives

Closely working with clinicians and in the context of translational research projects, disease map groups aim to make these resources practically useful and applicable to addressing diagnostic and treatment questions.

### Examples of disease maps applications for aiding functional interpretation

While the Disease Maps effort is comparatively new and we are only beginning to learn the power of the approach on a larger scale, there are several examples of the use of disease maps validated by experiments and/or using patients’ data.

#### Synergistic effect of combined treatment in cancer

**Fig. 5:**
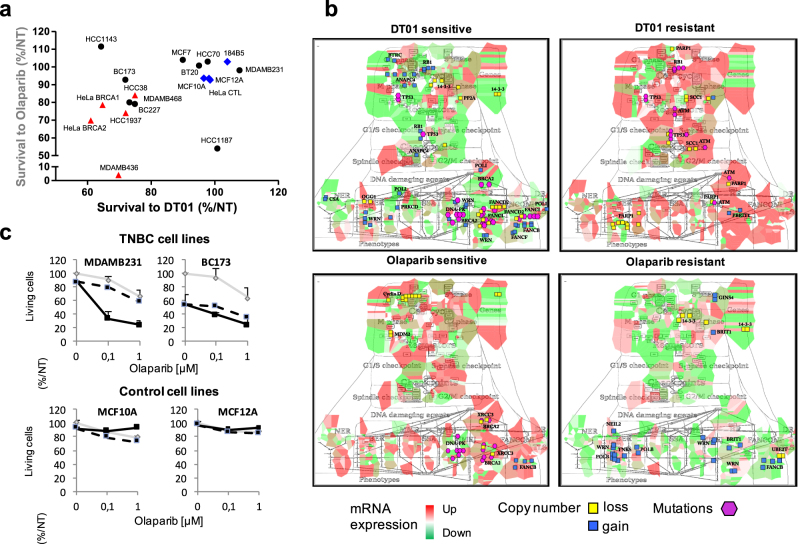
Sensitivity of TNBC cell lines to combination of DNA repair inhibitors. **a** Correlation analysis of survival to DT01 and Olaparib in TNBC and control cell lines. **b** Molecular portraits of DT01 and Olaparib-sensitive/resistant TNBC cell lines visualised on DNA repair map. **c** Cell survival to combination of DT01 and Olaparib. With DT01 (black line), without DT01 (grey line), dashed lines indicate calculated cell survival for additive effect of two drugs (adapted from Jdey et al.^42^)

An integrative analysis of omics data from triple-negative breast cancer (TNBC) cell lines showed that at least 70 non-overlapping genes were robustly correlated with sensitivity to DNA repair inhibitors Dbait (DT01) and Olaparib. Further analysis in the context of the ACSN maps demonstrated that different specific defects in DNA repair machinery were associated to Dbait or Olaparib sensitivity. Network-based molecular signatures highlighted different mechanisms for cells sensitive/resistant to Dbait and Olaparib, suggesting a rationale for the combination of these two drugs. The synergistic therapeutic effect was confirmed for the combined treatment with Dbait and poly(ADP-ribose) polymerase (PARP) inhibitors in TNBC while sparing healthy tissue^42^ (Fig. 5).

#### Finding metastasis inducers in colon cancer through network analysis

**Fig. 6:**
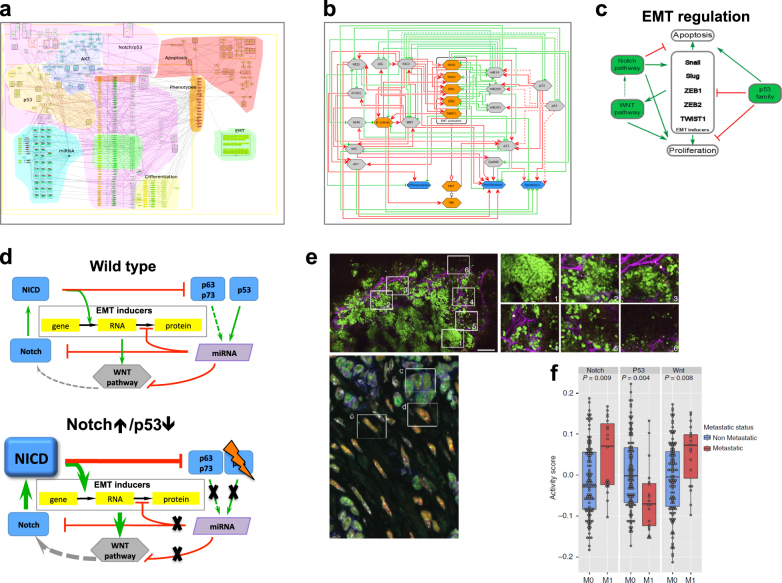
Prediction of synthetic interaction combination to achieve invasive phenotype in colon cancer mice model. **a** Comprehensive signalling network of EMT regulation, a part of the ACSN. **b** Scheme representing major players regulating EMT after structural analysis and reduction of signalling network complexity. **c** Simplistic model of EMT regulation. **d** Simplistic model explaining regulation involving Notch, p53 and Wnt pathways. **e** Lineage tracing of cell in tumour and in distant organs and immunostaining for major EMT markers. **f** Regulation of p53, Notch and Wnt pathways in invasive colon cancer in human (TCGA data) (adapted from Chanrion et al.^45^)

To study the interplay between signalling pathways regulating the epithelial-to-mesenchymal transition (EMT), the corresponding module was manually created and integrated into the ACSN. Next, the network complexity reduction was performed in BiNoM^43^ while preserving the core regulators of EMT. The reduced network was used for modelling EMT phenotypes resulting in the prediction that the simultaneous activation of Notch and the loss of p53 can promote EMT.^44^ To validate this hypothesis, a transgenic mouse model was created with a constitutively active Notch1 receptor in a p53-deleted background. EMT markers were shown to be associated with modulation of Notch and p53 gene expression in a manner similar to the mice model supporting the predicted synergy between these genes^45^ (Fig. 6).

It is important to note that the hypothesis driven from the disease map was not intuitive and actually contradicted the commonly accepted dogma in the colon cancer field. This clearly demonstrates that gathering cell signalling mechanisms together may lead to discovering unexpected relationships and new mechanisms.

Another example is available in Supplementary material S2: complex intervention gene sets are derived from data-driven network analysis for cancer patients in order to block “proliferation”’ phenotype, all the proliferation-inducing pathways have to be identified and assembled together, including alternative ones.^46^

### Future perspectives for hypothesis generation and opening new lines of investigation

Several directions are important for exploring further and carefully collecting and improving successful pipelines.

#### Visualisation of complex data in the context of networks

The ACSN has been used for defining precise case–control groups of patients, leading to their better stratification in cancer.^10^ Similarly, the Parkinson’s disease map has been used to interpret differentially regulated brain transcripts based on a clinical characterisation of patients.^46^ This stratified visualisation can be reinforced by enrichment analysis tools.^47,48^ A generic methodology would allow us to determine the biological process and pathway patterns that correspond to distinct phenotypic patient subgroups in a manner that facilitates patient stratification.

#### Network analysis of disease-related mechanisms

Integrating high-throughput patient data with information about the underlying machinery has the potential to reveal molecular patterns specific to disease subtypes and inform combinatorial diagnostics or therapeutics.^49^ This enables the identification of a set of interactions, whose joint alteration can shift the state of the network from unfavourable toward the desired outcome. For instance, analysing disease map’s perturbations can help predicting sensitivity to drugs based on network topology and choosing a patient-specific combination of drug targets.^50^ Identification of condition-specific mechanisms can facilitate the identification of disease-specific and subtype-specific biomarkers, intervention points or candidates for drug repurposing. A comparative analysis allows understanding the relative importance of certain mechanisms, as in the case of the comparison of Parkinson’s disease and ageing-related networks.^51^

#### Mathematical modelling: from static to dynamic representations

A disease map is initially a static representation of current knowledge, a collection of integrated scenarios, each reflecting a certain stage of a disease or a disease subtype. To gain further insights into the dynamic aspects of pathobiology and disease progression, a disease map has to be transformed into a dynamic mathematical model. The type of applicable mathematical model depends on the quality and the level of detail of the knowledge represented in a given disease map. Transcriptional regulatory and signalling networks are well suited for logic models, e.g., deterministic and stochastic Boolean networks, or for rule-based approaches. Metabolic networks can lead to steady-state approaches such as flux balance analysis.^52^ When detailed kinetic data are available, quantitative chemical kinetic models can be developed.^53^

## Expected impact of the Disease Maps Project

The following elements outline the forthcoming development and anticipated outcomes of the community effort.

- Establishing dedicated trusted reference resources on disease mechanisms for many diseases. One disease or disease subtype—one resource. Enabling advanced data interpretation, hypothesis generation, hypothesis prioritisation.
- Each disease map is a reflection of the current conceptual model of what disease mechanisms are. Enabling various types of mathematical modelling and predictions.
- Cross-disease comparison: specific and common disease mechanism and corresponding biomarkers for better diagnostics. New possibilities for studying disease comorbidity.
- Enabling advanced systems pharmacology and suggesting drug repositioning.
- Redefining diseases and their subtypes based on molecular signatures (endotypes). Preconditioning the development of new-generation disease ontologies.
- Developing technologies and advancing research strategies in computational biology and related fields through promoting a modular approach, consolidating resources and avoiding duplicated activities, in particular for building and managing complex mathematical models and designing prototypes for clinical decision support systems.

## Conclusion

Our increasing knowledge of pathway networks and how they vary across diseases has created a challenge of scale that needs significant collaborative activity to address. The Disease Maps Project sets out to stimulate the development of the tools and infrastructure that will support the current and next generation of work in translational research. Covering a growing number of diseases means that more and more proteins, RNAs, genes and metabolites will be included into high-confidence expert-verified functional modules. In turn, this will lead to a better understanding of not only various diseases but also health and well-being.

We are convinced that, through the establishment of a multidisciplinary community with shared practices and standards, the Disease Maps Project will facilitate powerful advances in the discovery of disease mechanisms, cross-disease comparison, finding disease comorbidities, suggesting drug repositioning through the identification of the common pathways. After careful validation, disease ontologies can be redefined based on disease endotypes—confirmed molecular mechanisms—thus paving the way to more precise, cost-effective personalised medicine and health-care solutions.

### Data availability

All relevant data are available within the paper, Supplementary Information and the Disease Maps website at http://disease-maps.io.

## Electronic supplementary material

Supplementary Materials

## Electronic supplementary material

**Supplementary information** accompanies the paper on the *npj Systems Biology and Applications* website (10.1038/s41540-018-0059-y).
