# Author Correction: Systems medicine disease maps: community-driven comprehensive representation of disease mechanisms

**DOI:** 10.1038/s41540-025-00554-6

**Published:** 2025-07-12

**Authors:** Alexander Mazein, Marek Ostaszewski, Inna Kuperstein, Steven Watterson, Nicolas Le Novère, Diane Lefaudeux, Bertrand De Meulder, Johann Pellet, Irina Balaur, Mansoor Saqi, Maria Manuela Nogueira, Feng Q. HeFeng, Andrew Parton, Nathanaël Lemonnier, Piotr Gawron, Stephan Gebel, Pierre Hainaut, Markus Ollert, Ugur Dogrusoz, Emmanuel Barillot, Andrei Zinovyev, Reinhard Schneider, Rudi Balling, Charles Auffray

**Affiliations:** 1 European Institute for Systems Biology and Medicine, CIRI UMR5308, CNRS-ENS-UCBL-INSERM, Université de Lyon, 50 Avenue Tony Garnier, 69007 Lyon, France; 2 Luxembourg Centre for Systems Biomedicine (LCSB), University of Luxembourg, Campus Belval, 7 Avenue des Hauts-Fourneaux, L-4362 Esch-sur-Alzette, Luxembourg; 3 Institut Curie, Paris, France; 4 INSERM, U900 Paris, France; 5 Mines ParisTech, Fontainebleau, France; 6 PSL Research University, Paris, France; 7 Northern Ireland Centre for Stratified Medicine, Ulster University, C-Tric, Altnagelvin Hospital Campus, Derry, Co Londonderry, Northern Ireland BT47 6SB UK; 8 The Babraham Institute, Babraham Research Campus, Cambridge, CB22 3AT UK; 9 Department of Infection and Immunity, Luxembourg Institute of Health (LIH), House of BioHealth, 29 Rue Henri Koch, L-4354 Esch-Sur-Alzette, Luxembourg; 10 Institute for Advanced Biosciences, University Grenoble-Alpes-INSERM U1209-CNRS UMR5309, Site Santé – Allée des Alpes, 38700 La Tronche, France; 11 Department of Dermatology and Allergy Center, Odense Research Center for Anaphylaxis, University of Southern Denmark, Odense, Denmark; 12 Faculty of Engineering, Computer Engineering Department, Bilkent University, Ankara, 06800 Turkey

Correction to: *npj Systems Biology and Applications* 10.1038/s41540-018-0059-y, published online 02 June 2018

In this article the author name Feng Q. HeFeng was incorrectly written as Feng He. The original article has been corrected.

