# Supplementary material for: Systems medicine disease maps: community-driven comprehensive representation of disease mechanisms
Source: NPJ Syst Biol Appl. 2018 Jun 2;4:21. doi: 10.1038/s41540-018-0059-y (PMC5984630; doi:10.1038/s41540-018-0059-y)
Supplement: Supplementary file 1 — Supplementary Materials [file 41540_2018_59_MOESM1_ESM.pdf]

Supplementary material S1

The authors' experience of participating in large-scale translational research projects

U-BIOPRED, Unbiased Biomarkers for the Prediction of Respiratory Disease Outcomes
www.imi.europa.eu/content/u-biopred
www.europeanlung.org/projects-and-research/projects/u-biopred/home
2009 - 2015
Funded under: IMI (http://www.imi.europa.eu/)
Authors participated: Bertrand De Meulder, Diane Lefaudeux, Alexander Mazein, Charles Auffray

eTRIKS, European Translational Information & Knowledge Management Services
www.imi.europa.eu/content/etriks
www.etriks.org
2012 - 2017
Funded under: IMI (http://www.imi.europa.eu/)
Authors participated: Mansoor Saqi, Irina Balaur, Alexander Mazein, Bertrand De Meulder, Diane Lefaudeux, Charles Auffray

MeDALL, Mechanisms of the Development of Allergy
www.medalldatabase.com
2010 - 2015
Funded under: FP7-HEALTH
Authors participated: Nathanaël Lemonnier, Johann Pellet, Charles Auffray

PRECISE: Personalized Engine for Cancer Integrative Study and Evaluation
2015 - 2018
http://precise-project.eu/
Funded under: EU Horizon 2020
Authors participating: Andrei Zinovyev, Emmanuel Bartillot

COLOSYS: A systems approach to preventing drug resistance in colon cancer
2015 - 2018
http://www.colosys.org
Funded under: EU ERACoSysMed-1
Authors participating: Inna Kuperstein, Andrei Zinovyev, Emmanuel Bartillot

Agilent Thought Leader Award
2013 - 2016
http://sysbio.curie.fr/projects.html
Funded under: Agilent Thought Leader Award to the "Computational Systems Biology of Cancer" team in Curie Institut
Authors participated: Inna Kuperstein, Andrei Zinovyev, Emmanuel Bartillot

APO-SYS: Apoptosis systems biology applied to cancer and AIDS. An integrated approach of experimental biology, data mining, mathematical modelling and molecular medicine
2008 - 2012
http://cordis.europa.eu/project/rcn/88080_en.html
http://cordis.europa.eu/result/rcn/56809_en.html
Funded under: EU FP7-HEALTH
Authors participated: Andrei Zinovyev, Emmanuel Bartillot

ASSET: Analysing and Striking the Sensitivities of Embryonal Tumours
2010 - 2016
http://www.ucd.ie/sbi/asset
Funded under: EU FP7-HEALTH
Authors participated: Andrei Zinovyev, Emmanuel Bartillot

ESBIC-D: European systems biology initiative for combating complex diseases
2005 - 2008
http://cordis.europa.eu/publication/rcn/12855_en.html
http://cordis.europa.eu/result/rcn/51789_en.html
Funded under: EU FP6
Authors participated: Andrei Zinovyev, Emmanuel Bartillot

SHIVA trial: Toward a therapeutic decision based on tumour molecular profile NCT01771458
2012 - 2015
https://siric.institut-curie.org/news/faits-marquants/shiva-first-randomised-precision-medicine-trial?_ga=1.136781616.1038784660.1371629844
Funded under: French national grant
Authors participated: Andrei Zinovyev, Emmanuel Bartillot

Supplementary material S2

Complex intervention gene sets derived from data-driven network analysis for cancer patients

Targeted therapies seem to be particularly promising for cancer treatment, though novel questions have arisen concerning tumour resistance during treatment with these novel drugs. A possible explanation is that the signal could eventually find an alternative pathway leading to cell division. A computational strategy was developed for identifying such alternative pathways based on the analysis of a detailed reaction network of MAPK pathway: 1) identification of tumour stage-specific active modules compared to normal cells using GSEA method[1] and 2) computation of optimal intervention points, whose disruption blocks all the proliferative pathways[2]. Minimal hitting sets (blocking sets with no blocking subsets) can be computed using the OCSANA algorithm, which optimises the trade-off between the number of components to disrupt and the potential side effects on the final global phenotype[3]. The procedure was applied to five different bladder tumour stages. For each of them, differential gene expression levels were computed using publicly available high-throughput expression

data for a cohort of bladder tumours[4]. To block the 'proliferation' phenotype, players from each pathway should be tucked together[2] (Figure S1).

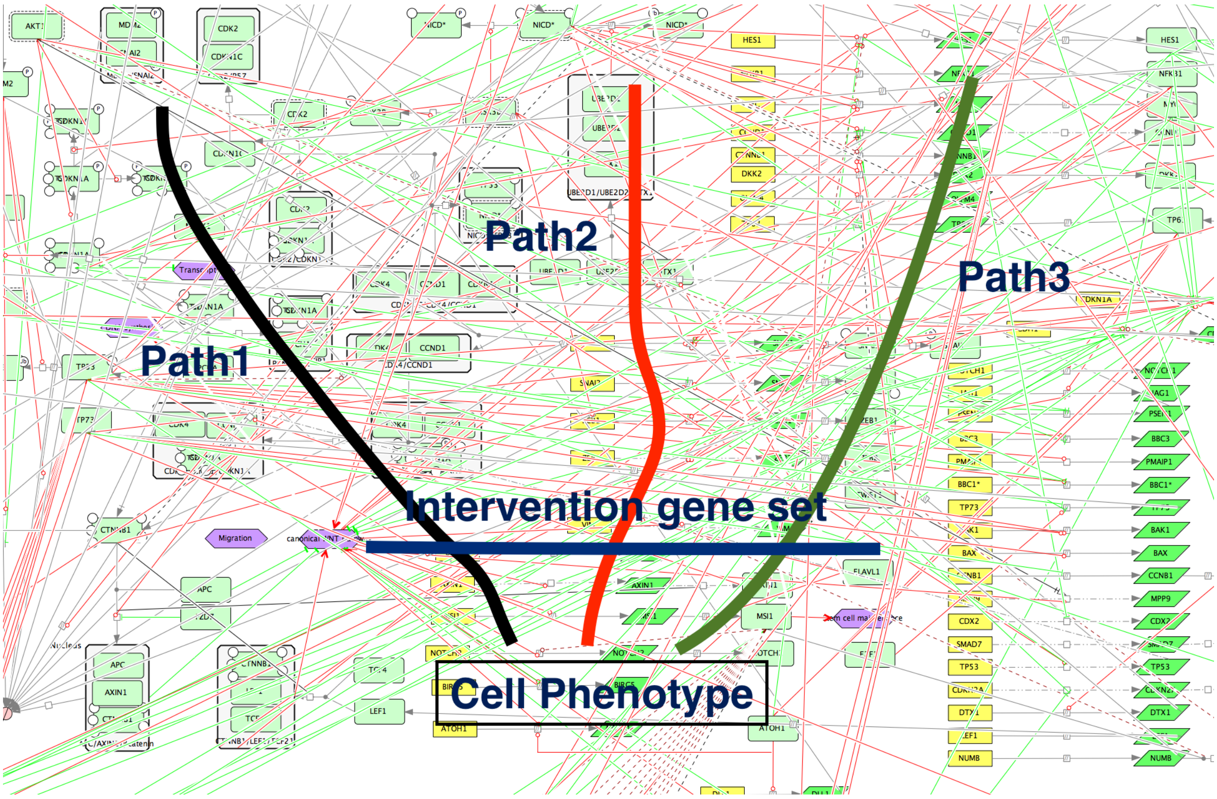

Figure S1. Schematic representation of intervention gene set principle. Various pathways in the signalling network contribute to a phenotype. To block phenotype, players from each pathway should be tucked together[2].

References

1. Subramanian, A. *et al.* Gene set enrichment analysis: a knowledge-based approach for interpreting genome-wide expression profiles. *Proc. Natl. Acad. Sci. U. S. A.* **102,** 15545–15550 (2005).
2. Kuperstein, I. *et al.* The shortest path is not the one you know: application of biological network resources in precision oncology research. *Mutagenesis* **30,** 191–204 (2015).
3. Vera-Licona, P., Bonnet, E., Barillot, E. & Zinovyev, A. OCSANA: optimal combinations of interventions from network analysis. *Bioinforma. Oxf. Engl.* **29,** 1571–1573 (2013).
4. Stransky, N. *et al.* Regional copy number-independent deregulation of transcription in cancer. *Nat. Genet.* **38,** 1386–1396 (2006).
